# Total-tau in cerebrospinal fluid of patients with multiple sclerosis decreases in secondary progressive stage of disease and reflects degree of brain atrophy

**DOI:** 10.3109/03009734.2012.669423

**Published:** 2012-08

**Authors:** Jacek Jaworski, Marek Psujek, Marzena Janczarek, Małgorzata Szczerbo-Trojanowska, Halina Bartosik-Psujek

**Affiliations:** ^1^Department of Neurology, Medical University of Lublin, Poland; ^2^2nd Department of Anaesthesiology and Intensive Care, Medical University of Lublin, Poland; ^3^Department of Interventional Radiology and Neuroradiology, Medical University of Lublin, Poland

**Keywords:** Brain atrophy, multiple sclerosis, tau protein

## Abstract

**Introduction:**

Tau protein is a potential marker of neuronal damage. The aim of the study is to investigate its potential role as a marker of brain atrophy in multiple sclerosis (MS).

**Materials and methods:**

Cerebrospinal fluid (CSF) and blood samples were collected from 48 patients with multiple sclerosis. Total-tau (t-tau) and phospho_181Thr_-tau (p-tau) concentrations were assayed with commercially available INNOTEST® hTAU Ag and INNOTEST® phospho181Thr-tau_(181P)_ and correlated with indices of brain atrophy in magnetic resonance imaging (MRI) and clinical characteristics of the study population.

**Results:**

T-tau concentration in CSF was significantly higher in relapsing-remitting (RR) compared to secondary progressive (SP) MS patients (*P* = 0.01). Brain parenchymal fraction (BPF) was significantly decreased in SP patients (*P* = 0.002). BPF in the whole study population correlated inversely with Expanded Disability Status Scale (EDSS) (*r* = –0.51, *P* = 0.0002) and Multiple Sclerosis Severity Score (MSSS) (*r* = –0.42, *P* = 0.002). T-tau in CSF in the whole patient group correlated inversely with EDSS (*r* = –0.58, *P* = 0.0006).

**Conclusions:**

The results of our study suggest that total-tau concentration in CSF in a MS population decreases in the course of disease and reflects degree of parenchymal brain loss.

## Introduction

Tau protein was historically described as a factor facilitating microtubule assembly *in vitro* (1). The protein belongs to a family of microtubule-associated proteins and is primarily localized in axonal and to a lesser extent in dendritic processes of neurons (2). Its acknowledged physiological role is to stabilize microtubules and thus maintain shape and structural polarization of neurons (3). As tau protein is regarded a possible marker of neuronal damage, a number of studies have been carried out to find a rationale for its clinical use in various neurological conditions.

Multiple sclerosis (MS) is a chronic autoimmune inflammatory disease of the central nervous system of unknown origin, primarily involving white matter and causing axonal degeneration (4). One of the important issues in the pathogenesis of MS is brain atrophy that constitutes substrate for irreversible neurological disability. Brain atrophy can be seen from the time of the first clinical presentation and progresses in the course of the disease (5). Despite decent theoretical background supporting the use of tau protein as a disease marker, research on cerebrospinal fluid (CSF) tau concentration in multiple sclerosis has so far yielded contradictory results. Some authors confirm the possible role of tau protein as a disease marker (6–8), whereas others deny its utility (9,10).

An algorithm developed in 2005, the Multiple Sclerosis Severity Score (MSSS) (11), allows assessment of disease severity by combination of single Expanded Disability Status Scale (EDSS) (12) and disease duration. The latest article (13) confirms the role of MSSS in the assessment of disease severity over time.

Magnetic resonance imaging (MRI) allows visualization of demyelinating lesions in the central nervous system and was incorporated in MS diagnostic criteria (14). The importance of this technique is enhanced by the fact that, beside clinical symptoms, it became the second modality to confirm the pivotal assumption of MS diagnostics, i.e. dissemination of symptoms in time and space. Apart from direct brain imaging, MRI allows measuring brain and spinal cord atrophy that thus helps to appreciate the degree of central nervous system degeneration, being the final consequence of MS pathological process. On the other hand, novel techniques, like MR spectroscopy, MR tractography, and magnetic tensor imaging, provide additional data on MS pathophysiology (15).

Our working hypothesis was that the tau protein concentration in CSF reflects the degree of brain atrophy in a population of MS patients with relapsing-remitting (RR) and secondary progressive (SP) disease types.

The aim of our study was to correlate indices of brain atrophy in brain MRI (brain parenchymal fraction, total brain volume, and CSF volume) with CSF and serum levels of total-tau (t-tau) and phospho181Thr-tau (p-tau) proteins. Additionally, T1 hypointense lesion (‘black holes') and fluid attenuated inversion recovery (FLAIR) hyperintense lesion volumes were calculated and correlated with indices of brain atrophy, levels of tau proteins, and clinical characteristics of the study population.

## Materials and methods

CSF and blood samples were collected from 48 patients referred to our clinical ward with suspected demyelinating disease. Multiple sclerosis was diagnosed according to the McDonald criteria (14) with further revisions (16). Female to male ratio was 27:21. Patients were categorized as having an RR course in 34 cases (71%) (female:male, 22:12) and an SP course in 14 cases (29%) (female:male, 5:9). Age, duration of disease, EDSS, and global MSSS are given in Table I. The study was approved by the local ethics committee of the Medical University of Lublin. Patients gave their informed consent to participate in the study, and samples were drawn as a part of a routine neurological work-up in a hospital setting.

**Table I.** Clinical characteristic of the study population.

	All patients	RR	SP
Age (mean ± SD) in years	36.2 ± 11.4	32.8 ± 9.1	44.5 ± 12.2
Range	21–62	21–52	21–62
Disease duration (mean ± SD) in years	6.4 ± 5.5	4.6 ± 4.0	10.8 ± 6.1
Range	1–28	1–18	2–28
EDSS (mean ± SD)	3.6 ±1.8	2.7 ± 1.0	6.0 ± 1.2
Range	1–7.5	1–4.5	4–7.5
MSSS (mean ± SD)	6.0 ± 2.1	5.3 ± 1.8	7.8 ± 1.8
Range	0.7–9.6	0.7–8.6	2.8–9.6

EDSS = Expanded Disability Status Scale; MSSS = Multiple Sclerosis Severity Score; RR = relapsing-remitting; SP = secondary progressive.

Blood samples were collected in polypropylene tubes and completely coagulated. Afterwards, they were centrifuged at 2000 *g* for 10 min. Clear serum was frozen at –80°C and stored for further assays. Lumbar punctures were performed in the morning to avoid diurnal variations in protein concentrations. A spinal catheter was introduced through the L3/L4 or L4/L5 interspace, and 5 mL of clear CSF were drawn. Samples were then centrifuged at 2000 *g* for 10 min, and supernatants were frozen in polypropylene tubes at –80°C for further examination. Samples were thawed just prior to laboratory processing. Total-tau (t-tau) and phospho_181Thr_-tau (p-tau) levels were assayed in duplicate with usage of commercially available enzyme-linked immunosorbent assay (ELISA) kits purchased from Innogenetics (Ghent, Belgium): INNOTEST® hTAU Ag and INNOTEST® PHOSPHO-TAU_(181P)_ for t-tau and p-tau, respectively. The t-tau assay was designed to detect both phosphorylated and non-phosphorylated tau residues. The lowest detection threshold for total-tau was 60 pg/mL. The p-tau assay was designed to detect only molecules phosphorylated on threonine_181_. The lowest detection threshold was 15.6 pg/mL. ELISA procedures were performed at room temperature. For both assays the absorbance was measured with spectrophotometer at 450 nm. The standard blank was a sample of diluent alone. Intra-assay variability was <10%. T-tau and p-tau were assayed by an independent operator following instructions provided in the kit inserts.

In case t-tau protein was not detectable, measurement was rejected from further analysis. However, if t-tau protein level was detectable but below the threshold values mentioned above, we assumed lowest detection threshold for further analysis.

Brain MRI was acquired as a part of routine hospital management with a Picker Eclipse GE Healthcare 1.5 Tesla scanner. MR T1-weighted images were acquired with gradient echo sequence, with TR = 300 ms and TE = 4 ms, flip angle = 80°. MR FLAIR images were acquired with spin echo sequence, with TR = 13,716 ms, TE = 112 ms, and TI = 1,800 ms, flip angle = 90°. In-plane resolution in all sequences was 1 mm, and slice-thickness was 5.0 mm.

A first MRI examination was carried out in all patients at baseline simultaneously with serum and CSF sampling. In 25 patients brain MRI was performed twice after a mean period of 3.6 years (SD = 2.3).

Brain morphometric measures were performed with Java Image (JIM) 5.0 software from Xinapse Systems with the use of ‘brain finder' and ‘multiple sclerosis lesion finder' tools, using axially orientated images.

The ‘brain finder' tool was used to calculate brain volume, CSF volume, and brain parenchymal fraction (BPF). BPF was defined as the ratio of parenchymal volume to the total volume within the brain surface contour:

$$\mathrm{BPF}=\frac{\mathrm{parenchymal}\;\mathrm{volume}}{\begin{array}{l} \mathrm{parenchymal}\;\mathrm{volume}+\;\mathrm{CSF}\;\mathrm{volume} \end{array}}$$

After initial brain delineation, images were checked, and brain border was manually corrected where needed. Brain volume was calculated on the basis of pixel intensity. Each set of images was analysed visually to ensure appropriate calculation. Threshold pixel intensity, distinguishing brain from CSF, was set manually in all images to 300.

Lesion load was calculated on T1-weighted and FLAIR MRI brain images with the ‘MS lesion finder' tool. Initially, during visual inspection, lesions were marked. A point marker was used in cases of well demarcated lesions. In cases of lesions with less distinct borders, the ‘irregular region of interest' marker was used. Before lesion volumes were calculated, the most appropriate settings were checked by trial and error. Finally, ‘weight on prior probabilities' was set to 0.6, and fuzzy threshold was set to 0.25. Lesion volume was calculated with use of ‘connect in 3D' option.

All statistical calculations were accomplished with GraphPad Prism 5.01 software. Normality was assessed using the D'Agostino–Pearson test. In cases where normality could not be assumed, relations between variables were calculated as Spearman correlation coefficient (*R*), and group comparisons were performed with the Mann–Whitney *U* non-parametric test; values were then presented as medians and range. If normality was assessed unpaired Student's *t* test was adopted, Pearson coefficient (*R*), was used, and values were presented as means and standard deviation. Linear regression coefficient (*R^2^*) with 95% CI was used to present graphical relations between variables in the Figures. *P* < 0.05 was regarded as statistically significant.

## Results

T-tau in CSF was detectable in 31/48 of cases (64.6%). P-tau in CSF was detectable in 48/48 of cases (100%). Total-tau in serum was detectable in 16/48 of cases (33%). P-tau in serum was detectable in 41/48 of cases (85%). Due to the low detection rate of t-tau in serum, these measurements were not included in further analysis.

T-tau and p-tau concentrations in CSF, p-tau concentration in serum, brain volumes, CSF volumes, and BPF for RR and SP patients are summarized in Table II.

**Table II.** Summarized results of CSF and serum tau protein concentrations and brain morphometric measurements of the study population.

	All patients	RR	SP
CSF t-tau in pg/mL (median)	105	110	68
Range	60–305	60–305	60–205
CSF p-tau in pg/mL (median)	35.1	38	30
Range	15.6–103	15.6–103	16–78
Serum p-tau in pg/mL (median)	29.2	27.1	33.5
Range	15.6–472.3	15.6–115.3	15.6–472.3
Brain volume in mL (mean ± SD)	1317 ± 165.6	1332 ± 160	1279 ± 179
CSF volume in mL (mean ± SD)	214.8 ± 55.6	198 ± 38	256 ± 71
Brain parenchymal fraction (mean ± SD)	0.86 ± 0.04	0.87 ± 0.03	0.83 ± 0.04

CSF = cerebrospinal fluid; t-tau = total-tau; p-tau = phospho181Thr-tau; pg/mL = picograms per mL; RR = relapsing-remitting; SP = secondary progressive.

**Figure 1.**
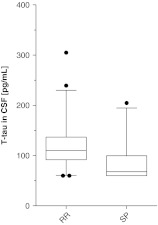
CSF total-tau concentrations in different MS populations (box represents median with interquartile 25th–75th range; whiskers show 10th–90th percentile range; dots show values beyond defined percentiles). Mann–Whitney *U* *P* = 0.01. (CSF = cerebrospinal fluid; t-tau = total-tau; pg/mL = picograms per mL; RR = relapsing-remitting; SP = secondary progressive).

T-tau concentration in CSF was significantly different in the RR and SP groups (Mann–Whitney *U P* = 0.01) with higher values in the RR group (Figure 1). No difference was found with respect to p-tau in CSF, nor in serum between the RR and SP groups. BPF was significantly higher in RR patients compared to the SP group (Student's *t* test *P* = 0.002). CSF volume differed significantly, with higher values in the SP group (Student's *t* test *P* = 0.006). There was no significant difference between the RR and SP groups regarding brain volume.

EDSS was significantly lower in the RR group compared to SP patients (Student's *t* test *P* < 0.0001). A similar relation was detected with respect to MSSS which was significantly lower in RR patients (Student's *t* test *P* = 0.0001).

**Figure 2.**
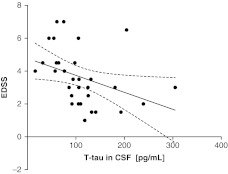
Negative correlation of t-tau with EDSS in the whole study population (*y* = –0.01*x* + 4.8) (linear regression line with 95% CI, *R^2^* = 0.14, *P* = 0.04; Spearman *R* = –0.58, *P* = 0.0006). (EDSS = Expanded Disability Status Scale; t-tau = total-tau; pg/mL = picograms per mL).

T-tau in CSF in the whole patient group correlated inversely with EDSS (Spearman *R* = –0.58, *P* = 0.0006; linear regression *R^2^* = 0.14, *P* = 0.04) (Figure 2).

**Figure 3.**
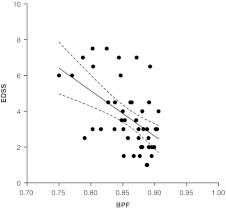
Negative correlation of EDSS with BPF in the whole study population (*y* = –25*x* + 25) (linear regression line with 95% CI, *R^2^* = 0.26, *P* = 0.0002; Pearson *R* = –0.51, *P* = 0.0002). (BPF = brain parenchymal fraction; EDSS = Expanded Disability Status Scale).

**Figure 4.**
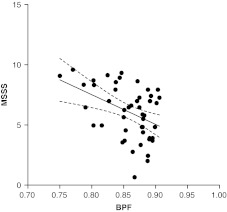
Negative correlation of MSSS and BPF in the whole study group (*y* = –25*x* + 27) (linear regression line with 95% CI, *R^2^* = 0.18, *P* = 0.002; Pearson *R* = –0.42, *P* = 0.002). (MSSS = Multiple Sclerosis Severity Score; BPF = brain parenchymal fraction).

BPF in the whole study population correlated inversely with EDSS and MSSS. For EDSS: Pearson *R* = –0.51, *P* = 0.0002; linear regression *R^2^* = 0.26, *P* = 0.0002 (Figure 3). For MSSS: Pearson *R* = –0.42, *P* = 0.002; linear regression *R^2^* = 0.18, *P* = 0.002 (Figure 4).

**Figure 5.**
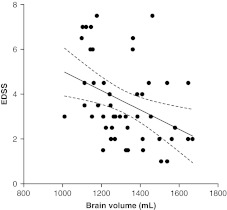
Negative correlation of brain volume with EDSS in the whole study population (*y* = –0.004*x* + 9.4) (linear regression line with 95% CI, *R^2^* = 0.15, *P* = 0.007; Pearson *R* = –0.38, *P* = 0.007). (EDSS = Expanded Disability Status Scale).

Brain volume (in mL) in the whole study population correlated inversely with EDSS (Pearson *R* = –0.38, *P* = 0.007; linear regression *R^2^* = 0.15, *P* = 0.007) (Figure 5).

In the follow-up period of mean 3.6 years, in the population of 25 patients:

1. BPF decreased significantly from a mean value of 0.87 (SD = 0.026) to 0.84 (SD = 0.049), *P* = 0.0008.
2. Brain volume (mL) decreased significantly from mean value of 1,351 (SD = 172) to 1,286 (SD = 176), *P* = 0.001.
3. CSF volume (mL) increased significantly from mean value of 198 (SD = 32) to 245 (SD = 69), *P* = 0.01.

BPF decrement correlated with time of observation (Pearson *R* = 0.63, *P* = 0.0008; linear regression *R^2^* = 0.39, *P* = 0.0008).

Brain volume (mL) decrement correlated with time of observation (Pearson *R* = 0.71, *P* < 0.0001; linear regression *R^2^* = 0.51, *P* < 0.0001).

CSF volume (mL) increment correlated with time of observation (Pearson *R* = 0.56, *P* = 0.004; linear regression *R^2^* = 0.31, *P* = 0.004).

EDSS correlated positively with T1-weighted lesion load (Spearman *R* = 0.46, *P* = 0.0004; linear regression *R^2^* = 0.22, *P* = 0.0007) and FLAIR lesion load (Spearman *R* = 0.4, *P* = 0.004, linear regression *R^2^* = 0.15, *P* = 0.007).

BPF did not correlate with T1-weighted lesion load or with FLAIR lesion load.

FLAIR lesion load was significantly higher than T1-weighted lesion load (median values were 11.07 mL versus 4.00 mL; Mann–Whitney *U P* = 0.0002) in the whole study population. There was a trend to show higher T1-weighted and FLAIR lesion load in the SP group compared to RR patients, but this difference did not reach statistical significance.

## Discussion

Tau protein belongs to a group of neuron-derived proteins that appear in CSF exclusively due to a process of diffusion from neuronal cytoplasm. Tau diffuses along CSF pathways, from the ventricular system to the lumbar region, along the diffusion gradient. Tau protein is then absorbed (similarly to CSF) in subarachnoid spaces through arachnoid granulations and spinal nerve roots into dural venous sinuses (17). Experimental works performed by Reiber showed that the tau concentration in the brain ventricular system is approximately 1.5 times higher than in the lumbar region. Continuously the lumbar CSF:serum total-tau concentration ratio is 10:1 (18). These differences in concentration force diffusion flow between brain and CSF, and along CSF pathways. The data on tau levels in various compartments significantly influence the interpretation of our results as tau was checked in serum and CSF from the lumbar region.

Low detection of total-tau (64.6% and 33% in CSF and serum, respectively), compared to its phosphorylated on threonine_181_ moiety (100% and 85% in CSF and serum, respectively), can be easily explained by different detection thresholds assumed in available ELISA kits. In the work by Reiber mentioned above (18), the total-tau assay was modified to increase sensitivity. This modification led to a better detection of tau protein and confirms that the inability of our assay to detect total-tau is not due to absence of this protein in CSF or in serum but reflects methodological drawbacks.

Tau protein can be detected in CSF and serum of healthy individuals of different ages without any apparent central nervous system (CNS) pathology (18–20). As the only reasonable source of tau protein is the neuronal cell, tau must be released from neuronal cytoplasm and enter the extracellular fluid and therewith CSF. This phenomenon cannot be explained exclusively by subclinical forms of dementia, as the population assayed for tau immunoreactivity ranged between 21 and 93 years (20). Tau leakage occurs most probably spontaneously and in this context may be regarded as a marker directly reflecting number of neurons in a brain. Thus, CSF tau concentration in healthy individuals presumably reflects a dynamic balance between tau release and clearance.

Moreover, we can assume that different pathological mechanisms result in various t-tau leakage patterns. In acute conditions, like stroke (21,22), head trauma (23,24), and viral encephalitis (25), t-tau enters CSF rapidly, reflecting neuronal damage, but undergoes fast clearance and thus can be detected shortly after an event. In these cases, increased concentration was observed exclusively in terms of t-tau but not p-tau, suggesting direct neuronal damage. In the course of chronic conditions with presumably membrane protein and intracellular pathology, like Alzheimer's disease (AD) and Creutzfeldt–Jakob disease (CJD), tau is constantly released into the CSF and therefore able to be continuously detected. Degree and dynamics of neuronal pathology explain higher CSF tau concentrations in CJD (26,27) compared to AD (28,29). Additionally in AD, pathologically phosphorylated tau protein forms intraneuronal inclusions (neurofibrillary tangles) which are postulated a cause of increased p-tau in CSF (30). However, the extensive neuronal damage seen in CJD, reflected by significantly higher t-tau concentrations in CSF, is not accompanied by a comparable increase in p-tau concentration (as neurofibrillary tangles are not a mainstay of CJD pathology) (31).

On the contrary, the RR type of MS is an autoimmune inflammatory disease with a pathological process driven by lymphocytic attack on myelin sheaths producing oligodendrocytes in the CNS. Demyelination with relative axonal preservation is the main pathological process. Despite clear evidence showing axonal loss being present from an early stage of the disease, basic MS pathophysiology remains extracellular (32). During the RR stage the inflammatory process predominates, but as MS enters secondarily a progressive stage, the inflammatory process is overwhelmed by neurodegeneration with continuous accumulation of neurological deficit (33). This is supposedly the main reason for contradictory results concerning tau concentrations in MS. Presumably, different conclusions were drawn on the basis of different populations. In acute relapse tau is released from compromised axonal processes and diffuses into CSF pathways. However, restoration of axonal integrity during the recovery that follows acute relapse stops tau leakage. Tau is then cleared from CSF, and its concentration resumes normal values. As a consequence, comparisons of MS population with normal subjects may not show any difference in CSF tau concentration, provided that MS patients have the potential to recover (as in RR stage) or the degree of CNS damage is limited. In fact a number of studies confirmed similar CSF t-tau concentrations between MS and control groups (9,10). It is worth noticing that in these studies SD for t-tau mean values was 2–3 times higher compared with SD in healthy controls. The meaning of this issue is that the MS population was not homogeneous and included patients with different disease activity which could significantly influence the final results. Analysis of other studies which found significant differences in CSF t-tau between MS patients and controls again shows comparable variability in t-tau levels (6–8). Heterogeneity of the MS population was noticed in a study by Kapaki et al. (6), which showed a significant difference in CSF t-tau levels between MS patients and controls. In this work (6), the CSF t-tau concentration in MS patients demonstrated bimodal distribution; however, two populations could not be distinguished on the basis of available clinical factors.

In the SP stage, with predominant neurodegenerative mechanisms, the constant decrease in neuronal density results in loss of tau resources; thus a lower t-tau in CSF reflects degree of brain atrophy. A number of studies confirmed progressive neurodegeneration in the course of MS that begins in the early stage of the disease. BPF was found to be lower in MS patients compared to healthy controls and to decrease continuously in the course of the disease (34). In magnetic resonance proton spectroscopy studies, N-acetylaspartate (NAA) levels (a neuronal marker) in normal white matter were decreased in MS patients compared to healthy controls, and the difference was more pronounced in the progressive stage (35,36). A recent study of untreated MS patients showed that brain atrophy proceeds in the course of MS, apparently independently of disease subtype (37).

In AD, continuous brain atrophy is accompanied by an increase of CSF t-tau and p-tau concentrations. The negative relation was strengthened in the context of regional cortical atrophy for t-tau (30), but also whole-brain atrophy for both t-tau and p-tau (38). However, the pathological process in AD, driven by accumulation of extracellular amyloid deposits and formation of intraneuronal neurofibrillary tangles is almost absent in MS patients before 64 years of age, as was proven in a neuropathological study (39). As none of our patients exceeded 64 years of age, we cannot assume that AD pathology influenced our results. Lack of AD pathology in our MS population serves as one possible explanation for the different relation of tau protein to brain atrophy, i.e. loss of brain volume with the increase of t-tau in CSF in AD, and the decrease of t-tau in CSF in MS.

The most striking result of our study is the significant difference between CSF t-tau concentrations between RR and SP patients, with higher values in the RR group (Figure 1; Table II). To our best knowledge this observation has not yet been published.

Difference in t-tau concentration is consistent with BPF values, showing decrease of parenchymal fraction in the course of MS, thus indirectly reflecting degree of brain atrophy. Difference in BPF can be attributed to an increase in intracranial CSF volume, reflecting loss of parenchymal tissue. Accordingly, the degree of disability expressed in EDSS and disease severity in MSSS is higher in SP patients compared to RR population, and correlated inversely with BPF. Negative correlation of CSF t-tau with a degree of motor disability in EDSS merges theoretical assumptions, forming a pattern in which t-tau indirectly reflects degree of neuronal loss.

Additional calculations performed on the basis of follow-up brain MRI in 25 patients confirmed BPF and brain volume decrement in the course of MS. Our study showed that the degree of disability (EDSS) correlated with T1-weighted and FLAIR lesion load. This result is confirmatory of previously published studies (40,41).

In our material T1-weighted lesion load was significantly lower than FLAIR lesion load. This observation can be explained on the basis of the pathophysiological importance of various lesion types. Lesions appearing as T1 ‘black holes' represent irreversible neuronal loss and appear late as a result of profound parenchymal damage (41). A recently performed study found that this type of lesion correlated well with degree of disability (42). On the contrary, FLAIR T2 hyperintense lesions may represent partially reversible damage and thus can be detected more frequently. One of the longitudinal studies estimating T2 lesions showed that approximately one-third of T2 lesions consisted of transient hyperintensities with no persistent signal change (43). In one recent study the t-tau concentration was used as a possible marker of cognitive decline in MS. T-tau concentration was correlated with number and volume of T2 lesions, including gadolinium enhancing lesions, in brain MRI, but no significant correlation was observed (44), similarly to our results. However, this study did not incorporate measurements of whole brain or regional brain atrophy. Additionally, in the result section there is no information on the number or the volume of T1-weighted lesions, possibly because the authors concentrated on inflammatory mechanisms influencing cognitive functions. Anyway, this report gave another proof for various types of pathology in MS compared to AD, as no significant difference was found in t-tau levels between MS and controls.

In conclusion, the results of our study suggest that t-tau concentration in CSF in MS population decreases in the course of disease and indirectly reflects the degree of parenchymal brain loss. The influence that a disease subtype exerts on the t-tau concentration in CSF depends apparently on the predominant pathophysiological process (neurodegeneration in the SP stage and inflammation in the RR stage). It is important to note that our results are population-based. Due to a number of conceivable factors influencing the tau concentration in CSF and the relatively small concentration of this protein in CSF of MS patients (which is in most cases comparable to the normal population), a single tau assay is not enough as an individual biomarker. Anyway, population measurements confirm constantly on-going neuronal loss in the course of MS and thus emphasize the need for future studies in search of MS aetiology and effective treatment.
